# Recent advances in the antepartum management of diabetes

**DOI:** 10.12688/f1000research.15795.1

**Published:** 2019-05-08

**Authors:** Cristina Mitric, Jade Desilets, Richard N Brown

**Affiliations:** 1 Obstetrics & Gynaecology, McGill University, Montreal, Quebec, H4A 3J1, Canada

**Keywords:** Pregnancy, diabetes, gestational diabetes, macrosomia, perinatal outcomes

## Abstract

Gestational and pre-gestational diabetes are frequent problems encountered in obstetrical practice and their complications may influence both the mother (such as hypertension, pre-eclampsia, increased caesarean rates) and the foetus (such as macrosomia, shoulder dystocia, respiratory distress, hypoglycaemia, or childhood obesity and diabetes). Given the important implications for mothers and their offspring, screening and appropriate management of diabetes during pregnancy are essential. This is a review of articles published between 2015 and 2018 on Medline via Ovid that focus on advances in the management of diabetes in pregnancy. Recent data have concentrated predominantly on optimising glycaemic control, which is key for minimising the burden of maternal and foetal complications. Lifestyle changes, notably physical exercise and diet adjustments, appear to have beneficial effects. However, data are inconclusive with respect to which diet and form of exercise provide optimal benefits. Oral glycaemic agents—in particular, metformin—are gaining acceptance as more data indicating their long-term safety for the foetus and newborn emerge. Recent reviews present inconclusive data on the efficacy and safety of insulin analogues. New technologies such as continuous insulin pumps for type 1 diabetes and telemedicine-guided management of diabetes are significantly appreciated by patients and represent promising clinical tools. There are few new data addressing the areas of antenatal foetal surveillance, the timing and need for induction of delivery, and the indications for planned caesarean section birth.

## Introduction

Hyperglycaemia during pregnancy is a common condition associated with maternal and foetal adverse outcomes such as pre-eclampsia, macrosomia, shoulder dystocia, increased risk of stillbirth, and neonatal hypoglycaemia ^1, 2^. Gestational diabetes mellitus (GDM) is defined as any degree of glucose intolerance that is first diagnosed during pregnancy, whereas pre-gestational diabetes is defined as diabetes mellitus (DM) (type 1 or 2) present before conception ^3^. The incidence of diabetes has been increasing worldwide, and the prevalence of hyperglycaemia, as defined by the 2013 World Health Organization (WHO) diagnostic criteria, is estimated to be as high as 16.6% during pregnancy; GDM represents 84% of these cases ^3, 4^.

Given the important maternal and foetal complications, identifying and optimally treating diabetes during pregnancy are of paramount importance. The goal of this review is to highlight new evidence in the antepartum management of hyperglycaemia during pregnancy. A search and review of articles published between 2015 and 2018 on Medline via Ovid were conducted and salient points were summarised. The article will discuss glycaemic surveillance and control using non-pharmacological and pharmacological methods as well as advances in the obstetrical management in the antepartum period.

## Diagnosis

Although the need to screen for GDM is universally accepted, the approach through which this should be achieved remains contentious. The International Association of Diabetes and Pregnancy Study Groups (IADPSG) recommendation of a single-step 75-g oral glucose challenge test (OGTT) screening strategy has been adopted by the WHO ^3^. However, because this approach is perceived to result in an increase in GDM prevalence, many organisations have persisted with a two-step approach. In 2016, considering recommendations of the Canadian Diabetes Association (now known as Diabetes Canada), the Society of Obstetricians and Gynaecologists of Canada (SOGC) endorsed a two-step screening approach with an initial 50-g glucose challenge test (GCT) for all pregnant women ^2^. Although the American College of Obstetricians and Gynecologists (ACOG) recommends screening all women, the choice of approach and cut-off values are not standardised, but a two-step approach is favoured ^5^. The Royal Australian and New Zealand College of Obstetricians and Gynaecologists (RANZCOG) suggests screening all women but is against using a two-step approach and instead advises direct use of a 75-g OGTT ^6^. In contrast, the Royal College of Obstetricians and Gynaecologists/National Institute for Health and Care Excellence (RCOG/NICE) advises screening only women with risk factors for GDM using a single step 75-g OGTT ^7^.

The most recent of many Cochrane reviews addressing the best approach determines that there are still insufficient data to conclude which approach is best and that only large-volume well-conducted randomised control trials (RCTs) will resolve this ^7^. One recent study has evaluated the use of a two-step approach using the 2013 WHO adopted criteria and this has not supported the continuing use of a 50-g GCT ^8^. However, the benefits of using the WHO criteria, which have increased the prevalence of GDM some four-fold over the rate previously diagnosed with the two-step approach, need a more robust prospective evaluation. First-trimester screening for pre-existing hyperglycaemia presents an even greater dilemma. The glycaemia threshold used to identify women who will benefit from early intervention is not known. The concept of early GDM, as opposed to abnormal results being interpreted as indicative of pre-existing DM, is increasingly recognised, but there is a paucity of data to define this ^9^.

## Glycaemic control

### Glycaemia monitoring and treatment target

Maternal hyperglycaemia is associated with adverse maternal and foetal outcomes and there is a well-established association between increasing glycaemia and the occurrence of adverse outcomes ^2, 10^. Although control of glycaemia during pregnancy has been shown to reduce adverse maternal and neonatal outcomes, no absolute threshold at which adverse risks occur has been identified. The most recent societal guidelines in glucose monitoring and glycaemia targets are reported in Table 1 ^2, 10– 14^.

Although therapy adjustment based on postprandial blood glucose levels is associated with improved outcomes ^15^, there are currently no standardised criteria regarding the precise steps that should be taken to optimise glucose control. A recent meta-analysis identified RCTs which used various levels of strictness for intensifying glucose control, ranging from a single recorded value exceeding target values to more than 50% of recordings being above target values, and concluded that there is not enough evidence to recommend one approach over the other given the wide variations seen across the included studies ^16^. A retrospective study by Scifres *et al*. suggests that hyperglycaemia in gestational diabetes might require a tighter control in the obese population, in whom the effects of hyperglycaemia on pregnancy seem amplified ^17^. The use of continuous glucose monitors in pregnancy has been gaining popularity, although the data are sparse; an underpowered RCT showed inconclusive benefits with this monitoring method ^18– 21^.

**Table 1.** Societal guidelines regarding glucose monitoring and target glycaemia in pregnancy.

	Canadian Diabetes Guidelines (2018) and Society of Obstetricians and Gynecologists of Canada (2016)	National Institute for Health and Care Excellence (2015 and 2016)	American College of Obstetricians and Gynecologists (2016 and 2018) and American Diabetes Association (2018)	International Federation of Gynecology and Obstetrics (2015)
Timing of measurement	Fasting blood glucose Post-prandial (three times)	Fasting blood glucose 1 h post-prandial	Fasting blood glucose Post-prandial Pre-prandial ^a^	Fasting glucose Two or three times 1 to 2 h post-prandial ^b^
Target glycaemia, mmol/L	Fasting and pre- prandial < 5.3 1 h post prandial < 7.8 2 h post prandial < 6.7	Fasting < 5.3 1 h post prandial < 7.8 2 h post prandial < 6.4	Fasting < 5.3 1 h post prandial < 7.8 2 h post prandial < 6.7	Fasting < 5.3 1 h post prandial < 7.8 2 h post prandial < 6.7

^a^Pre-prandial measurement recommended for some women with pre-pregnancy diabetes. ^b^Daily measurement if in a low-resource setting.

### Non-pharmacological: lifestyle changes

Lifestyle changes represent the first-line approach to therapy in gestational diabetes and include dietary modification and physical activity with the aim of limiting gestational weight gain and improving glycaemic control. Although there is still controversy regarding optimal gestational weight gain, a retrospective study by Wong *et al*. found no difference in obstetrical outcomes with restricting weight gain beyond the 2009 Institute of Medicine criteria for patients with gestational diabetes ^22^. Lifestyle modification alone is sufficient in about 70 to 85% of women with diagnosed GDM to achieve glycaemic targets ^11^. Although most guidelines recommend a 1- to 2-week trial of lifestyle modification, pharmacotherapy should not be delayed, as euglycaemia is important in reducing adverse outcomes ^2, 10, 11, 23– 25^. To date, there is inconclusive evidence as to when to initiate pharmacotherapy in cases of failure of the first-line approach; however, the conclusions of a meta-analysis suggest that pharmacotherapy should be considered in women with GDM when one or two glucose values exceed target levels at 1 or 2 hours postprandial during a 1- or 2-week trial period ^24^.

Most international obstetrical associations advocate for an immediate referral to a certified dietician and increased physical activity at the time of diagnosis of GDM ^2, 10, 25^. A Cochrane review evaluated the impact of lifestyle modifications on weight gain and showed less gestational weight gain, decreased risks of macrosomia and caesarean delivery but no impact on incidence of pre-eclampsia or preterm birth ^26^. There is evidence showing a beneficial effect of a low glycaemic index diet, but more studies are required to define precisely what a low glycaemic index diet should entail ^25, 27, 28^. Although diet is the cornerstone of treatment, good data are lacking; previous limited-power RCTs show a benefit with low-carbohydrate, high-vegetable and whole-grain diets ^29, 30^. Small studies suggest that carbohydrate restriction may be associated with unintended adverse effects ^31, 32^. Other dietary approaches with probiotics and vitamin supplements have gained popularity, but the evidence is insufficient to recommend their generalised use ^33, 34^. One RCT with 140 patients with GDM showed that co-supplementation with vitamin D and fatty acids was associated with lower glycaemia; however, maternal and foetal outcomes were not evaluated ^35^. Larger RCTs comparing different dietary approaches are still required before guidelines on the use of supplements can be developed.

A Cochrane review has evaluated the role of exercise in pregnancy on glycaemic control. Exercise was associated with lower fasting and postprandial blood glucose values but remained inconclusive with respect to long-term maternal or foetal effects ^36^. In addition, the data were insufficient to evaluate what form of exercise was most beneficial. Therefore, future studies will be required to validate and assess the efficacy and safety of standardised exercise regimens, especially since data on the safety of exercise in the first trimester are scarce ^37^. Nonetheless, in the absence of contraindications, physical activity, in combination with dietary changes, can be encouraged as an integrated part of the non-pharmacological approach.

## Pharmacotherapy

### Oral glycaemic agents

Although most national obstetrical associations continue to recommend insulin as first-line pharmacotherapy for diabetes in pregnancy given its inability to cross the placenta ^5, 11, 25^, certain oral glycaemic agents are gaining attention. For instance, the NICE in the UK recommended metformin as a first-line treatment in its 2015 guidelines, except in cases where the fasting plasma glucose level exceeds 7.0 mmol/L at diagnosis ^10, 38^. Meanwhile, Diabetes Canada (formerly the Canadian Diabetes Association) describes metformin as a promising glycaemic agent given its side effect profile and efficacy ^25^, and the medication is gaining ground in Australian obstetrical practice ^39^.

Several meta-analyses have studied the efficiency of metformin, showing its superiority to insulin in terms of reducing the risk of foetal hypoglycaemia, large-for-gestational-age foetuses, pregnancy-associated hypertension, and maternal gestational weight gain ^38, 40– 42^. Data suggest that between 14 and 50% of cases treated with metformin will require additional insulin to reach the target blood glucose levels, making it difficult for any meta-analysis to evaluate the effect of metformin alone given the frequent use of additional insulin ^41, 42^. To date, very few studies have evaluated the impact of metformin use during pregnancy on long-term maternal and foetal health ^40, 43^. One RCT (n = 97) found that children exposed to metformin in the prenatal period were heavier and taller at 18 months of age and had similar body compositions and no differences in social or linguistic development compared with controls ^44^. Another RCT (n = 146) found no differences in neurodevelopmental outcomes at 2 years of age between toddlers with *in utero* exposure to metformin versus those exposed only to insulin ^45^. This RCT also showed no difference in offspring body fat percentage at 2 years, although several skinfold measures were larger in metformin-exposed offspring ^46^. A further follow-up found similar total and abdominal body fat percentages at 7 to 9 years of age and no differences in metabolic measures between the offspring of mothers who received either metformin or insulin in pregnancy. However, in one subgroup population, children of mothers who received metformin were larger on several measures at 9 years of age than those who received insulin ^47^. These data, though somewhat reassuring, highlight the need for further investigation in this area.

In meta-analyses comparing oral pharmacotherapy in the treatment of GDM, glyburide is associated with higher birth weights and rates of macrosomia when compared with other agents, making its use less favourable ^38, 48, 49^. In the meta-analysis by Farrar *et al*., glyburide was estimated to be most effective in reducing caesarean section rate but less effective than metformin or insulin for other adverse outcomes related to GDM ^41^. When compared with insulin, glyburide appears to have worse neonatal outcomes, including more hypoglycaemia, macrosomia, birth injuries, and respiratory distress syndrome, and no improvement in glycaemic control ^49, 50^. Given these conclusions, glyburide should not be considered as a first-line treatment but rather should be held in reserve in cases where neither insulin nor metformin is tolerated or in cases where metformin is insufficient to control the glycaemia ^5, 25^.

Overall, oral glycaemic agents, particularly metformin, appear to be efficient in treating diabetes during pregnancy, but they do cross the placenta and the long-term effects on the foetus are not yet well defined. This information needs to be conveyed to the parents if an oral glycaemic agent is chosen ^11, 40, 43^.

### Insulin

When glycaemic control does not meet pregnancy goals with lifestyle changes, insulin is added as an adjuvant therapy. Recent Cochrane reviews have found no evidence to recommend one specific insulin type or regimen over any other in pregnancy ^51, 52^. Although insulin analogues are gaining clinical ground ^53^, the data to support their use are sparse. Specifically, the above Cochrane reviews have limited results on the benefits and safety of newer analogues, including glargine, lispro, and detemir ^51, 52^. Another meta-analysis concluded that there is a lack of information on the efficacy and safety of rapid-acting analogues lispro and aspart. A literature review found no association of lispro, aspart, or detemir with increased congenital anomalies compared with human insulin ^54^.

Limited review data illustrate that continuous insulin infusion pumps, though increasingly popular, offer no maternal or foetal advantages or disadvantages over the traditional multiple daily injection approach ^55^. For type 1 DM, the closed-loop insulin delivery approach has been shown to provide better glycaemic control over sensory-augmented pump therapy in an initial study of 16 patients; however, data on the efficacy, safety and feasibility of closed-loop therapies during pregnancy are lacking ^56, 57^. Although this new regimen appears to be well perceived by mothers with type 1 DM ^58^, additional larger RCTs are required to evaluate the effects of this approach on maternal and foetal outcomes.

### eHealth medicine

The use of information technology and web platforms for pregnant women with diabetes is rapidly increasing worldwide ^25^. Examples of such approaches are web uploads of capillary blood glucose measurements on cell-phone apps ^59^, apps which include lifestyle and dietary counseling ^60^, or even clinical decision support systems which suggest insulin adjustments based on glycaemic values ^61^. Telemedicine allows for prompt management of care across distances with fewer face-to-face medical visits ^62^ and has been associated with high patient satisfaction ^63– 65^. In 2016, Ming *et al*. published a meta-analysis of seven RCTs that involved telemedicine in gestational diabetes [62]. The authors showed similar maternal and neonatal outcomes such as glycaemic control, caesarean rates, macrosomia, and neonatal intensive care admissions, concluding that the evidence at the time was insufficient to show that telemedicine in gestational diabetes results in improved clinical outcomes. This was believed to be due to underpowered studies and the heterogenicity of e-platforms ^66^. A randomised study by Mackillop *et al*. included 208 patients with gestational diabetes followed via traditional glycaemic control or via an app and found that the app group had more satisfaction with care, better glycaemic control, lower incidence of preterm delivery, fewer caesarean section births, and similar costs ^64^.

Therefore, the use of e-platforms in gestational diabetes management shows promising results with respect to patient satisfaction and no detrimental effect on pregnancy outcomes. Whether such healthcare tools are cost-effective or can help improve care in urban or remote areas remains to be determined by adequately powered RCTs.

## Obstetrical approach

### Antenatal surveillance

Gestational and pre-gestational diabetes are associated with an increased risk of stillbirth ^2^ and therefore represent a population that requires more antenatal surveillance. The perfect surveillance strategy is not known and as such there are slight variations amongst societal guidelines, as illustrated in Table 2 ^2, 5, 10, 12– 14, 25^. No recent developments have been reported in the literature.

**Table 2.** Societal guidelines on timing and type of foetal antenatal surveillance in pregnancies complicated by diabetes.

	American College of Obstetricians and Gynecologists (2016 and 2018)	Society of Obstetricians and Gynecologists of Canada (2016)	Canadian Diabetes Guidelines (2018)	National Institute for Health and Care Excellence (2015 and 2016)	International Federation of Gynecology and Obstetrics (2015)
Pre-gestational diabetes	32 to 34 weeks	36 weeks	34 to 36 weeks	38 weeks	No specific recommendations
Gestational diabetes on diet	No specific recommendations		No specific recommendations		
Gestational diabetes on medication	32 weeks		34 to 36 weeks		
Type of surveillance	Bi-weekly NST for pre- gestational diabetes, daily kick count, and AFI	Growth US at 28 weeks, then every 2 to 4 weeks; NST, AFI, or BPP or a combination of these	Weekly NST, AFI, or BPP or a combination of these	US for growth and AFI every 4 weeks: 28 to 36 weeks	US every 2 to 4 weeks from diagnosis until term, NST, BPP, and kick count as per local protocol

AFI, amniotic fluid index; BPP, biophysical profile; NST, foetal non-stress test or foetal heart rate monitoring; US, ultrasound.

### Induction of labour

Given the concerns related to the increased risks of stillbirth, macrosomia, caesarean section and shoulder dystocia in pregnancies complicated by diabetes, there is ongoing discussion as to whether earlier induction would be beneficial in this patient population and, if so, at what gestational age. The timing of induction varies amongst obstetrical organisations and their specific recommendations are illustrated in Table 3 ^2, 10, 12– 14^. In 2017, the GINEXMAL trial randomly assigned 425 patients with low-risk gestational diabetes to induction of labour at 38 + 0 to 39 + 0 weeks versus expectant management until 41 weeks. Of note, they excluded patients with an estimated foetal weight above 4000g or with an unfavourable cervix. There was no difference in the rates of caesarean section (12.6% in induction group versus 11.8% in the expectant group, *P* = 0.81) or foetal or maternal morbidities other than increased rates of hyperbilirubinemia in the newborns in the induction group ^67^. A separate retrospective cohort study found that routine induction of labour at 38 or 39 weeks in women with gestational diabetes was associated with a lower incidence of caesarean section and a higher incidence of neonatal intensive care unit admission when induction was prior to 39 weeks ^68^. A Cochrane review published in 2018 included only the GINEXMAL trial and as such concluded that there is insufficient evidence regarding benefits of induction in gestational diabetes ^69^. In terms of mode of delivery, caesarean section is recommended above 4500g by the American College of Obstetricians and Gynecologists ^5, 12^, whereas for the International Federation of Gynecology and Obstetrics the threshold is 4000g ^14^.

**Table 3.** Societal guidelines on timing of induction of pregnancies complicated by diabetes.

	Society of Obstetricians and Gynecologists of Canada (2016)	American College of Obstetricians and Gynecologists (2016 and 2018)	National Institute for Health and Care Excellence (2015 and 2016)	International Federation of Gynecology and Obstetrics (2015)
Pre-gestational diabetes	38 to 40 weeks	40 weeks ^a^	37 to 38+6 ^a^	38 to 39 weeks if >3800 g or LGA ≤3800 g or AGA but poor compliance or control, previous stillbirth, or vascular disease
Gestational diabetes on diet		After 41 weeks	40+6	
Gestational diabetes on medication		39 to 39+6 ^a^	40+6 ^a^	

^a^Earlier deliveries to be considered if poor glycaemic control or maternal or foetal concerns. AGA, appropriate for gestational age; LGA, large for gestational age.

## Conclusions

Important information regarding the optimal management of diabetes in pregnancy is still emerging. This review illustrates some encouraging advances, including the use of oral hypoglycaemic agents—in particular, metformin—and insulin analogues. Diabetic tools such as continuous glucose monitoring and closed-loop insulin delivery show promising outcomes in small populations of patients with type 1 DM, whereas e-health technologies, such as online platforms for glycaemic monitoring, show encouraging results as modern approaches to glucose management. There is no new strong evidence to advocate for any significant changes in the existing recommendations for antenatal surveillance and labour induction.

## Abbreviations

DM, diabetes mellitus; GCT, glucose challenge test; GDM, gestational diabetes mellitus; NICE, National Institute for Health and Care Excellence; OGTT, oral glucose challenge test; RCT, randomised control trial; WHO, World Health Organization
